# Notes of a Recent Visit to Several Provincial Asylums for the Insane in France

**Published:** 1850-10-01

**Authors:** John Webster


### NOTES OF A RECENT VISIT TO SEVERAL PROVINCIAL ASYLA FOR THE INSANE IN FRANCE.

BY JOHN WEBSTER, M.D., F.R.S., ETC.

Having taken considerable interest in promoting the study of mental diseases, and endeavoured to direct professional attention to that important subject, particularly in reference to the advantages of rendering the public institutions of London—appropriated for the reception and care of persons labouring under insanity, more available than heretofore to medical students, anxious to investigate the nature and study the treatment of that interesting class of maladies, I was induced, during past years, to pay various visits to the Parisian lunatic hospitals, in order to see the system pursued in regard to this point, at the great establishments of the French metropolis. Although well satisfied with the information then acquired, yet feeling desirous of farther examining the provincial French asyla for the insane, more at leisure, than previous tours enabled me to make, I accordingly visited several of these establishments during last autumn; and being much interested with many things which came under observation, I am therefore led to believe the short notes cursorily made during my peregrination, may now, perhaps, prove interesting to some of the readers of the Psychological Journal. Trusting such anticipations are not altogether groundless, even should some of the remarks appear discursive, I nevertheless hope the facts narrated will be deemed worthy of perusal, and not unsuitable to the pages of a publication which deservedly has much influence; whilst it is also specially dedicated to the investigation of diseases affecting the human mind, and the management of asyla intended for the treatment of patients labouring under the greatest of all afflictions—mental alienation.

Previous, however, to entering upon the subjects proposed for discussion in my present communication, it may be instructive to premise, according to the law of the French legislature, dated the 30th June, 1838, each department in France is obliged to provide a public establishment, destined for the reception and treatment of lunatic patients belonging to the district; or to arrange, under the sanction of the Minister of the Interior, with a public or private asylum in the same or a neighbouring department, to receive their insane paupers; or, in certain cases, to appropriate a separate division, in civil hospitals, for lunatics, provided there be sufficient accommodation for not less than fifty patients. As every lunatic establishment is placed under the direction of the Préfet of the Department, the President of the Tribunal, the Procureur of the Republic, the Judge of the Peace, and the Mayor of the Commune; and as they must be visited by the Procureur of the Arrondissement, at least every six months (in addition to the visits made by the Préfet, and the other official persons delegated by him, or by the Minister of the Interior, for that purpose), there is some guarantee they will be properly conducted. But before any establishment can be opened for the admission of insane patients, besides these regulations, all rules for their internal administration must be first approved by the Minister. Again, by another clause of the same act, it is expressly forbidden for any person to establish, or even to superintend a private insane asylum, without the authorization of government; and in such cases, it is also enacted, that every house, intended for the reception of lunatic patients, must be entirely separate from any private establishment receiving inmates affected with other diseases; whilst the Procureur of the Arrondissement should visit each private asylum in the district, at least once every three months, at undetermined periods.

According to the subsequent ordonnance of the 18th December, 1839, which regulates many details not comprehended in the Act of 1838, it is also ordered, that every public asylum for the insane shall be administered under the authority of the Minister of the Interior, and the Préfet of the Department; who are to be assisted by a commission of five members, appointed by the Préfet, but acting gratuitously. The Director of the establishment, and the Physicians, are, in the first instance, nominated by the Minister; but if vacancies afterwards occur, the Minister must appoint from a list of three candidates proposed by the Préfet. However, the patronage in reality still remains with the Minister, as he may add certain parties, of his own free will, to the list of candidates, and then nominate the favoured protégé to the vacant office. Besides, as the Minister can revoke the appointments of director or physicians, upon the report of the Préfet; as he settles the amount of the salaries of these officers; and farther, as the Préfets are the servants of the Minister, by whom they are appointed, and at whose pleasure they retain their offices, the Minister of the Interior thus becomes the sole patron and dispenser of all the important appointments now attached to the public insane asyla in France; much in the same way as the Minister of Justice has the patronage of those in the law and legal tribunals. Although the physicians must reside, according to this ordonnance, within the asylum, some may, by favour, nevertheless, obtain a special permission from the Minister to live elsewhere; but in that case the party ought to visit the lunatics confided to his care, at least once every day, and should he be prevented doing so, this duty must be performed by another physician.

The above are some of the general regulations respecting public insane asyla in France; but when any person is desirous of obtaining a licence to open a private establishment, the applicant presents a petition to the Préfet of the Department in which the proposed asylum will be situated, to whose satisfaction the petitioner must prove that he is twenty-one years of age, and in the enjoyment of all his civil rights; that his conduct and morals have been good during the three previous years, as shown by a certificate from the Mayor of the Commune in which he has resided; and lastly, that he is a Doctor of Medicine. However, where the individual does not possess the latter qualification, he may produce an obligation from some physician who engages, with the Préfet's approval, to undertake the medical duties of, and to reside in, the asylum; and as the Préfet can, at any time, revoke this appointment, it is not likely the treatment of the patients will be much neglected. Further details respecting the constitution and government of the public and private insane establishments of France, might be given; but enough having already been said regarding the general administration of these institutions, I will only now add, that besides the official persons previously mentioned, there are also two Inspectors-General of all the lunatic institutions of the Republic, whose special duties, amongst others, are to visit and report upon these establishments to the Minister of the Interior; and as these responsible offices are now ably filled by M. Ferrus, formerly physician to Bicêtre, and M. Parchappe, lately physician to the asylum of Saint Yon, both very well known to the medical profession by their works on insanity; it is superfluous to speak of either of these gentlemen's qualifications for such important appointments.

Anterior to the first French revolution, the lunatic hospitals of that country, like other parts of Europe, then stood much in need of amelioration. The path first pointed out by the philanthropic Tenon, having been, however, zealously pursued by Pinel, that physician soon directed his benevolent mind to the treatment of insanity, and to improve the internal economy of those institutions, whereby great changes were effected, and the wretched condition of many insane patients greatly ameliorated. Subsequently, Esquirol, and other eminent persons, made the study of mental diseases, as also the moral and medical treatment of the insane, the subject of their particular inquiries, so that increased attention being bestowed upon these important questions, great improvements were made in the science and treatment of mania, not only in France, but throughout the entire civilized world.

Considering it would be superfluous, as also uninteresting, to extend the notes I propose transcribing to an unreasonable length, my remarks will therefore be limited to eight provincial French asyla recently visited; since, to attempt more, would render the present report unnecessarily voluminous. For similar reasons, I have also thought it advisable to refrain at present from alluding, except cursorily, to the public lunatic institutions of Paris, already so well known—namely, Bicêtre for males, and the Salpêtrière for females; which are the only public hospitals for insane pauper patients in the French metropolis. There is, however, the hospital at Charenton, for those of either sex who can pay a stipulated pension towards their treatment and maintenance.

Of these institutions, the Salpêtrière hospital is, perhaps, the largest establishment of the kind in Europe, being not only a workhouse for infirm women, but likewise an asylum for pauper insane females belonging to Paris and the environs. At present, the entire population of the Salpêtrière is about 5350, of whom nearly 1600 are insane and epileptic patients. To give some idea of the immense extent of this charitable institution, it may be stated, that upwards of 3000 pounds' weight of animal food are daily used in cooking, and in the kitchen, one of the largest in the world, the number of dinners daily prepared for the various inmates is often 5200; the materials of which are excellent. Bicêtre, like the Salpêtrière, is both a poor-house and an asylum for insane males belonging to the department of the Seine; but, although the entire population is under that of the Salpêtrière, it is always very considerable, the number of lunatics being seldom below 1200, including paralytic cases, epileptics, and idiots; the improvement of whose lamentable condition, through the skill and treatment of M. Voisin, has of late justly attracted much attention. Again, the asylum at Charenton, likewise a public establishment for lunatics, differs from Bicêtre and the Salpêtrière in two important particulars—viz., both sexes are admitted as patients, and they pay for their treatment and maintenance. This institution is administered under the authority of the Minister of the Interior; and the scale of payments for board embraces three classes. The first pay 1300 francs, the second 1000 francs, and the lowest 720 francs per annum; but the Minister may authorise the admission of gratuitous patients. Here, the total number of inmates varies from 450 to 500, of whom some belong to the highest orders in society.

Confining my notes, therefore, to asyla situated in the west and central departments of France, and for the reasons already stated, the first public institution I shall now bring under notice is that of

### Bon Sauveur, at Caen.

This hospice, besides being an asylum for lunatics of both sexes, is a religious establishment, containing a large population, which is thus enumerated in the official table given me by the authorities. 1st. The choir and lay-sisters consist of 237

individuals. 2nd. There are five priests, composed of the superior and four chaplains. 3rd. 26 free boarders, all resident; 20 being ladies, and 6 gentlemen. 4th. The deaf and dumb, of which there are 155, comprising 65 males, and 90 females. 5th. The resident domestics amount to 128, of whom 68 are males, and 60 females. And lastly, 692 lunatics; of whom 302 were men, and 390 women, at the period of my visit; thus making an aggregate population of 1243 persons, all living within the precincts of this institution. Besides the above numbers, there are also 2 physicians, and 80 non-resident work people; so that 1325 individuals now belong to this immense establishment.

This very important, and truly, one of the most remarkable institutions of France, is situated in the city of Caen, or rather, in one of its fauxbourgs, and not far from the old abbey of St. Etienne, which contains the tomb of William the Conqueror. It has a fine, if not an extensive prospect of the neighbouring country; still the situation is low, and I should consider somewhat damp, especially as the meadows immediately adjoining are flooded every winter. The governing body of the establishment is composed of "les Sœurs Religieuses," and the priests; the superior of whom is an autocrat, possesses supreme and irresponsible power in the management of the institution; whilst the order account to no one respecting their large revenue, expenses, or administration. This may in part be accounted for by the circumstance that its wealth and prosperity are mainly owing to the exertions of the clergy, and to former religious benefactors; particularly to the late Abbé Jamet, who endowed Bon Sauveur with all his property. This venerable ecclesiastic died in 1845, at the advanced age of eighty-three, having devoted his long life to promote the welfare of this his favourite establishment.

Besides the present hospice, the Order possesses two succursal institutions, one about thirty miles from Caen, the other near Albi, in the south of France; which last, I understood, is quite as important as that now described. Although the property of an ancient religious order, the surveillance of all lunatics resident within its walls rests, as in other French asyla, in the central government, who appoint the physicians charged with the treatment of every insane patient; still, the medical attendants' power is here not so defined as prevails in several institutions I could name, but of which more hereafter.

At the period of my visit to Bon Sauveur, the total number of lunatic inmates in the various divisions amounted to 692, as stated in a previous paragraph; of whom 302 were male, and 390 female patients; but of these, 212 women and 141 men pay for their board, lodging, and treatment, sums varying from 400 to 4000 francs annually; the remaining 339 inmates being all indigent persons. In reference to receiving private patients at this institution, it may be perhaps interesting to readers to mention, that the celebrated Beau Brummell died within the walls of Bon Sauveur; the apartment he occupied in this madhouse being shown me, where that quondam companion of George the Fourth was supported, during the last years of his chequered life, by the liberality of Mr. Armstrong, the late British consul at Caen.

Notwithstanding the numerous lunatic patients treated at this asylum, the medical staff consists of only two visiting physicians, Dr. Vastel taking charge of the male lunatics, and Dr. Faucon Duquesnay of the female department; the arduous duties of which they each perform regularly and zealously. But as neither of those experienced gentlemen reside at the asylum, both being practitioners of repute in Caen, and there are no internes, it hence follows, when any emergency occurs requiring medical aid, the attending medical officer must be sent for to his private residence. This is a very great defect, and I said as much to the authorities; but the holy sisterhood did not think it advisable to have pupils living within the walls of their convent, or even to allow a resident physician, although such is the strict law of the country, and as now carried out in most of the insane asyla throughout France.

During the year 1849, the movements of insane patients at Bon Sauveur was as follows:—

| | | | |
|---|---|---|---|
| Admitted ... ... ... | Males, 60 ... ... | Females, 63 ... ... | Total, 123. |
| Discharged cured... | Males, 42 ... ... | Females, 30 ... ... | Total, 72. |
| Died ... ... ... ... | Males, 31 ... ... | Females, 21 ... ... | Total, 52. |

Amongst the above deaths it should, however, be stated, that 18 men and 11 women died last year by cholera, whereby the mortality of the lunatics was considerably augmented beyond the ordinary average; and as similar effects from the recent epidemic will be noticed in the other lunatic institutions referred to in the present communication, this peculiar feature of the year 1849 must not be overlooked, when drawing conclusions respecting the ratio of deaths met with amongst the insane, in the various establishments I visited.

Not having been originally constructed for the reception of lunatics, the buildings are defective in many respects, especially the cells for the agitated patients, which have stone walls, iron bars, and unglazed windows; the beds in such localities being frequently wooden cages, into which the afflicted inmate is even locked at night, besides being tied to the bed, or confined by a strait waistcoat. The superior class of patients have, however, often excellent accommodation, some in detached houses, with gardens, particularly on the female side; whilst the gardens generally are beautiful, and kept in excellent order.

The sisterhood, and others attached to Bon Sauveur, have only occupied the present residence since 1805, when they first received insane men into their establishment; although previously, and even so early as 1728, they took charge of female lunatics, but more from charity than gain. However, considering the large number of paying inmates, amounting at present to 353 individuals, the sum now received by the executive must be considerable; and a director of an insane establishment in another part of France, who seemed as well acquainted with Bon Sauveur as he is also with the cost of keeping such institutions, told me distinctly the profit derived from the lunatics at this asylum, could not amount to less than 80 or 100,000 francs annually.

As in most French public asyla for the insane I have ever visited, the female lunatics in this hospice appeared much more agitated and noisy than the male patients; at the same time, mechanical restraint seemed oftener employed in the former than the latter sex. Thus, amongst the 390 insane females in the various wards on the day of my visit, 12 were in strait-waistcoats, some being also tied to seats or chairs, and one to a tree in the garden; besides which, three or four were very furious, and shut up in solitary cells, two of whom I noticed looking into the court-yard through a small opening made in the lower part of their cell door, the same as we usually find in English dog-kennels. This is no exaggeration, as I saw them with my own eyes, and also heard the poor sufferers howling within, and that even so recently as the month of August 1850! On the other hand, amongst the 302 male lunatics, only 3 were in strait-waistcoats, whilst three others had their arms restrained by leather straps. Besides these six patients, one man was also shut up in his iron-barred cell, who certainly made a very great noise, and thrust his clenched hands through the unglazed window as if to strike. This individual, the attendants said, was so dangerous, that they did not think it safe to approach within arms'-length of such an excited maniac.

Another male patient, who had recently committed murder, was likewise confined to his caged bed in a solitary cell; but this man was quiet, although very filthy. Referring again to the female wards, I would repeat, the inmates were exceedingly noisy; and when passing through a court-yard, in which the most agitated female patients were confined, being accompanied only by the attendant sisters, I must acknowledge our position then seemed somewhat dangerous; and although I have often perambulated similar departments in France, as well as in other countries, our sojourn here was far from agreeable; and my kind conductors thought so likewise, whilst they told me that one of their sisterhood had been almost strangled by a furious maniac then at large in the court-yard we had just visited.

So little importance seems attached at this institution to employing the strait-waistcoat in refractory cases, that I was informed, if any sudden fit of phrenzy seized a patient, the sisters in the ward, at their own discretion, would at once put the party in a camisole, or shut up the lunatic in one of the caged beds, to which they were likewise even bound by ligatures; and this was done, they said, as well for the patient's own safety as that of others, and even of the attendants'; the physician being, of course, duly informed of such proceedings at his subsequent visit to the institution.

Notwithstanding the remarks now made respecting the employment of mechanical restraint at Bon Sauveur, which I have now detailed from personal observation and inquiry on the spot, the benevolent sisters perform their painful duty amongst the many afflicted fellow-creatures by whom they are surrounded, most zealously and courageously, according to their own views respecting the nature and treatment of insane persons; but the system and machinery now in operation in such an establishment is bad; and I unhesitatingly assert, however well disposed or charitable the holy sisterhood are undoubtedly, there ought to be resident medical attendants, with internes, having the sole and responsible control over every patient; the entire management being also remodelled and made conformable to the recent laws enacted by the legislature, according to which every public lunatic establishment in France ought to be now governed.

Employment to a considerable extent is carried out, both amongst the male and female patients; the former being frequently occupied in cultivating the extensive gardens of the hospice, in the large drying-house, or in various trades and handicrafts; as also at a farm belonging to the establishment in the vicinity, where a portion of the insane patients are engaged in agricultural operations; and although axes in cutting wood, or other dangerous weapons, are often put into the hands of the lunatics, no harm or accident, I was informed, has ever resulted from such employments. Amongst the female patients, knitting, sewing, household work, and various other occupations, for which women are by nature adapted, were likewise zealously encouraged and put in practice; so much so, that it gives me infinite satisfaction to finish this brief notice of the Bon Sauveur Asylum, by saying, however much I differ from the authorities of this institution, respecting their frequent recourse to mechanical restraint, the endeavours constantly made to employ the insane patients are highly creditable and satisfactory.

When alluding to the holy sisterhood of this large institution, and the important position they still hold, it may be considered interesting to state, as an illustration of the persons often composing this religious body, that many belong to the upper ranks of society, of which the following is an instance. During my perambulations through the extensive apartments of the establishment, I was introduced to a noble "Comtesse" connected with a distinguished family, formerly attached to the court of one of the recent kings in France, when royalty was still in the ascendancy. This lady had also moved in the first circles of London, where she likely joined, as also at Paris, in the gaieties of fashionable life. However, here she now was, clad in the plain flowing black dress of her order, with her head enveloped in an ample snow-white hood; the only ornament she wore being a silver crucifix on the breast. Although no friend to monastic institutions, I could not but admire the devotion of this worthy individual, who had dedicated the remainder of her future existence and energies to attendance upon the sick, and to works of charity. That she had, nevertheless, not forgotten the world, her previous acquaintance, or the places she had formerly visited, was evident from our conversation; and amongst other subjects we talked of, and to which I was able to reply to her inquiries, one had reference to a distinguished physician, a friend of my own, now resident in London, of whom she spoke in complimentary language, having been attended by that gentleman when indisposed in the English metropolis. But similar examples to the above are even now not uncommon in France; and there is no class of persons who are so zealously disposed to dedicate their time and attentions to alleviate the miseries and bodily sufferings of their fellow-creatures, as many of these sisters of charity, who are often attached to the hospitals of this country.

### St. Meen's Asylum, near Rennes.

This lunatic institution is situated near the ancient capital of Brittany, upon a rising ground, about half a league from the city of Rennes. It possesses a good situation, apparently healthy, from its natural advantages; has a fine prospect of the neighbouring country, and is not overlooked by contiguous buildings. The house is, however, old, particularly one part; and this portion of the building, not having been constructed for an insane asylum, is not at all adapted for that purpose; indeed, one of the courts in the more modern quarter has been closed, because the female patients, who formerly there took out-of-door exercise, being overlooked by the male patients occupying the gallery above, the two sexes could thus converse together, which proved most improper and injurious. Even the best part of this structure looks more like a prison than a lunatic institution, having strong iron bars in the windows; and altogether it is not a proper receptacle for the insane, which seems also to be the opinion some of the managing authorities; whilst discussions had, I understood, actually taken place respecting the suppression of this establishment, and the removal of the patients elsewhere. From this and other causes, very little money was last year expended in repairs or improvements; and as differences of opinion prevailed, according to report, betwixt the local and central government in reference to its management, these circumstances only render the efforts made by the officials in the establishment, towards improving the well-being of the patients, more meritorious.

At present no resident physician is attached to St. Meen's, although there was one formerly. Since his removal by the Minister of the Interior to another asylum, the institution is only visited every other day by Dr. Pechot an eminent physician, in general practice at Rennes, appointed temporarily for that purpose, from his high qualifications and reputation. Two internes, however, reside in the house, but as both can never be absent at the same time, medical assistance is always at hand; and if any case of emergency occurs, the visiting physician is immediately sent for. Should he be otherwise engaged, or out of the way at the time, then one of the professors of the medical school is called in to see the case, and give directions. Farther, in order that no delay may ever occur under such circumstances, a horse and carriage is always kept ready to bring the physycian or deputy to St. Meen's; whilst on the days Dr. Pechot does not visit the asylum, an interne goes round the wards, sees all the patients, and prescribes whatever he thinks necessary.

The gardens of the asylum are extensive, and seem productive; and recently, there was also a farm attached to the institution, where the male lunatics were often employed; but this has been let to a tenant, whereby the agricultural occupations by patients has been interrupted, which is much to be regretted; however, hopes are entertained that the farm will be again resumed.

During the year 1849, the following was the movement of the insane patients at St. Meen's Asylum:—

| | | | |
|---|---|---|---|
| Admitted ... ... ... | Males, 47 ... ... | Females, 44 ... ... | Total, 91. |
| Discharged cured... | Males, 22 ... ... | Females, 15 ... ... | Total, 37. |
| Died ... ... ... ... | Males, 24 ... ... | Females, 25 ... ... | Total, 49. |

Of the 49 deaths now reported, it is necessary to mentiou, that 21 were caused by cholera, 10 of whom were men, and 11 women. Again, with reference to the total number of lunatics in the institution on the day I visited the wards, they consisted of 143 men and 168 women; making an aggregate population of 311 lunatics then under treatment.

This Asylum seemed more tranquil than others I had visited in France during previous years; especially on the female side of the house, the appearances in this respect being somewhat similar to an English institution for lunatics, and certainly very different from the female wards of Bon Sauveur. This feature of St. Meen's, which I could not help observing, may perhaps be owing to the more staid Breton character, compared with other French provinces; but whatever may be the cause, it was certainly not less remarkable, whilst, on the male side, there also reigned considerable tranquillity.

I saw no patients confined in their cells, and only two females were in strait-waistcoats; whilst three male patients were in camisoles, one of whom, however, I was informed, had been so kept for upwards of five years consecutively, excepting when locked up in his cell during the night, or occasionally left free for an hour or two in the day-time, to ease his limbs; but then he was always put into a cell for safety. No straps are used in this Asylum, and the strait waistcoat is the only instrumental restraint permitted, which measure must never be employed by any attendant without the specific order of the physician, or, in his absence, of the interne in charge of the patients; the great desire of all being to use restraint as seldom as possible; and doubtless this contributed to the marked quietude of the wards above observed.

Many of the patients were epileptics; several were affected with general paralysis, and some with erotomania; and what is somewhat singular, especially in France, three, if not four, could not be prevailed upon to speak at any time, and one of these, a man, had remained silent for years. Two female idiots also attracted my special attention, on account of the great mutual attachment these unfortunate sufferers exhibited. They were constantly together, slept in one bed, sat on the same bench, walked always with each other, ate at the same time, and they even laughed, or attempted to speak simultaneously; in short, the two were inseparable; and my informant added, he had long noticed this peculiarity.

The bodily health of the patients appeared in a satisfactory condition; they are well fed, comfortably clothed, and although the house was old, and inappropriate for lunatics, its management seemed, on the whole, creditable to the authorities. Every effort is made to employ the patients, both male and female; the men in the gardens, cutting firewood, weaving, shoemaking, carpenters' work, also in the kitchen, out-offices of the institution, and so forth. The women seemed especially busy in various occupations, which was greatly promoted by the zeal and perseverance of the chief female attendant—somewhat similar to the matron in an English asylum. This lady attached great importance to employing lunatics, as it materially promoted their recovery; and in consequence, nearly all the female patients in the wards seemed employed, either in spinning, knitting stockings, making clothes, mending, sewing, and similar occupations. Others were working in the kitchen, assisting in the bath-house, washing clothes, and cleaning apartments; in short, almost every female seemed more or less engaged, and as a proof that the labour of the patients was profitably promoted, the whole clothing in the establishment is made on the premises; whilst the inmates are encouraged to work, by giving them a certain proportion of the money they thus gain, as a recompence; whereby they may in the meantime obtain a few little comforts, and also possess some money, however small, when they leave the institution.

As in most other public asyla for the insane, private patients, who pay for their treatment and maintenance, are likewise admitted at St. Meen's; but owing to the uncertainty respecting the future fate of the establishment, and other causes, the number of that class of lunatics at present is inconsiderable; there being only 23 male patients and 19 females in the house, who pay from 500 to 1000 francs annually; and considering all things, the charges seemed fair and moderate.

Although a lunatic asylum, strictly speaking, and not a workhouse, like some other establishments, there are still a few patients in St. Meen's not insane, being only afflicted with cutaneous complaints. This exception is in compliance with an ancient regulation still in force; but the number of individuals of this description was very small when I visited the institution, and they are not enumerated in the statistical details given of the resident population; the 311 patients reported, being all more or less afflicted with mental diseases; many of whose maladies, as in most public French lunatic asyla, were of long continuance, and therefore incurable.

### Nantes Asylum.

This celebrated establishment, like that of Bon Sauveur, forms only one division of the " Hospice Géneral" of the ancient and populous city of Nantes. It is situated near the banks of one of the numerous branches of the Loire, in the fauxbourg of St. Jacques, on the south side of the river, having the water on one side, and a flat, open, yet richly cultivated country on the other. The gardens are extensive, very fruitful; and although the eye may be pleased with the external aspect of the buildings and locality, the situation is not well chosen, nor seems salubrious; and as the lunatic department forms only one division of an extensive charitable institution, having a large population, some of whom now labour under various forms of disease, whilst many others are paupers or infirm old persons; but especially as the entire establishment has only one large kitchen to supply the whole inmates, I consider the lunatic asylum, now under review, objectionably placed, notwithstanding the admirable arrangements of the building appropriated to the insane, which is one of the best throughout France; and may well be taken as a model for similar structures by public bodies, alone anxious to promote the comfort and proper classification of the insane, without looking too much to architectural ornaments, or ad captandum appearances.

The total population of the Hospice Géneral of Nantes, at the period of my visit, comprised 1196 individuals, who were thus distributed:—1st, 190 infirm old men, and 265 women of the same description, making a total of 455 inmates of what we call in England a workhouse. 2nd, 25 deaf and dumb persons. 3rd, 14 male and female boarders. 4th, 161 orphans or foundlings—composed of 82 boys and 79 girls; and lastly, 391 lunatics; to which must be added 150 individuals who compose the staff of officers, internes, sisters, nurses, teachers, and attendants of every description; thus making the aggregate number of 1196 persons, all resident in the institution.

Formerly, only one physician attended to the medical treatment of this large population; now, two practitioners of repute, resident in Nantes, and in general practice, have the medical charge of all the inmates excepting those who are insane; which important duty is confided to Dr. Bouchet, so well known in France as elsewhere, for his scientific attainments, and experience in mental diseases. The physician resides at the institution, and gives his whole time and attention to the insane patients of the asylum, being debarred from pursuing private practice. He has two internes, who reside in the house constantly; and if Dr. Bouchet considers it necessary, at any time, to have a consultation of physicians, or surgeons, especially in physical disease, or if an epidemic prevails amongst the inmates of the lunatic wards, he may call in any of the civil practitioners resident in Nantes he thinks advisable, who are paid by the administration of the hospice, for services so rendered, as if in private practice. This is an admirable arrangement, and worthy of imitation, particularly in cases where surgical operations are required.

During the year 1849, the following official report shows the movement of the patients in the lunatic department.

| | | | |
|---|---|---|---|
| Admitted ... ... ... | Males, 70 ... ... | Females, 56 ... ... | Total, 126. |
| Discharged, cured... | Males, 25 ... ... | Females, 25 ... ... | Total, 50. |
| Died ... ... ... ... | Males, 56 ... ... | Females, 44 ... ... | Total, 100. |

Amongst the 100 deaths above enumerated, cholera carried off 37 male, and 24 female lunatics; thus making 61 deaths by that epidemic malady; whereby the mortality of the insane inmates was considerably augmented above the ordinary average.

At the period of my visit, the total population of the division for lunatics amounted to 391 persons, as already stated, and was thus divided—Insane men, 181; women, 210: of whom 119 males, and 157 females were classed as indigent patients; whilst those who paid for their board, lodging, and treatment, consisted of 62 gentlemen, and 53 ladies; being altogether 115 persons, or nearly one-third of the entire number. The pensioners are divided into three classes, who pay from 750 to 1700 francs annually; but one female patient, belonging to the upper class of society, has a detached house and garden to herself, with servants, for which she pays 4000 francs per annum.

Having been constructed expressly for the reception of lunatics, the interior accommodation of the asylum is of a superior description; the dormitories are well ventilated; the court-yards judiciously arranged, and entirely separate; the one not overlooking the other, and having easy communication with the main galleries; whilst the attendants, from their own sleeping apartments, can easily inspect the various patients in each dormitory. Some of the private single sleeping rooms are perhaps rather small in dimension; nevertheless, the interior arrangements are better than in many other establishments, and the classification of the inmates appeared judicious. There are no iron bars in the windows; the safety of the inmates being assured by wire screens, the same as may be seen in ordinary houses, to prevent the glass from being broken. In the out-galleries overlooking the gardens, and in which the patients walk during bad weather, there are, however, iron bars between the stone pillars, to prevent accidents, which is a proper precaution.

The general aspect of the dormitories and court yards, when the lunatics were assembled, was quiet and orderly; although the female patients seemed rather noisy, as in most French asyla; but they were much less agitated than at Bon Sauveur. The number of patients under restraint, on the day of my visit, was twelve females—all in the strait-waistcoat, with nine males, two of whom had only their arms tied by straps, the hands being free, and the remaining seven in camisoles. In addition, one of the male patients so restrained, had also his legs tied together by hobbles, whereby he could only move very slowly through the court-yard. This patient was reported to be very dangerous, both to himself and others; and that cause was assigned as the reason for such treatment. No lunatic was otherwise prevented from locomotion, or taking exercise; and every cell was empty of tenants. In reference to the employment of the strait-waistcoat, it may be further mentioned, that all the male patients then under mechanical restraint were of the indigent class; but amongst the females thus treated, three were not of that description.

As elsewhere, many of the inmates were incurable patients, their disease being of long standing. One feature rather peculiar to this institution is, however, well worth recording—namely, the very small number affected with general paralysis, otherwise so common in France, especially in Paris, and upon which serious form of malady the French physicians have recently done much to illustrate; in this asylum the disease seemed of rare occurrence, only three cases of the kind being found amongst the whole 391 insane patients at present under treatment, two being males, and only one female.

Amongst the insane inmates, occupations of various kinds are carried on to a great extent in this asylum, many being employed in the gardens, or in different trades and handicrafts, unnecessary now to particularise; employing the mind diseased, if possible, through bodily labour, being considered one of the chief means of amelioration. Several were working in the smithy; some as carpenters, or shoemakers; others were digging in the gardens, or carrying away loads and assisting in forwarding the improvements still in progress. Amongst the females, many were engaged in sewing, knitting, making clothes, cooking, and in other household duties. Besides the above occupations, a small farm, of about thirty acres, being also attached to the hospital, the lunatics have thus a locality where they may be occupied in agricultural employments. But if I understood Dr. Bouchet's opinions correctly, that distinguished psychologist, although a decided advocate of out as well as in-door employments for lunatics, prefers horticultural to agricultural occupations; and in such a distinction I have heard other physicians also acquiesce.

In support not only of the advantages, but of the safety of employing lunatic patients in many ordinary occupations, although dangerous instruments may be even placed in their hands, it is most satisfactory to state, on the authority of such a witness as Dr. Bouchet, that during the last eighteen years he has been a zealous promoter of the labour system; no accident has resulted from so employing insane patients, excepting in a single instance a few years ago, when one of the lunatic labourers struck another inmate with a deal board he was then carrying towards another part of the premises. But on this point it may be reasonably asked; might not similar consequences follow, under the same circumstances, even amongst sane persons? The question is conclusive; whilst the idea of danger appears small, wherever the attendants adopt due precautions.

Before taking leave of the asylum at Nantes, it ought to be stated, that the system pursued in visiting the various patients, the minuteness and regularity adopted in all the details, in reference to their condition, treatment, and employment, struck me as being particularly worthy of imitation; whilst the registry of cases, the amount and quality of occupation performed by individual patients were particularly interesting; in short, I have no hesitation in saying, this institution deserves much of the praise it has already received from other observers, whether native or foreign.

### St. Gemmes Asylum, near Angers.

This provincial establishment for the reception of insane patients belonging to the department of the Maine et Loire, is situated in the village of St. Gemmes, on the neck of land which is bounded on the one side by the Maine, and on the other by the river Loire. It is built upon a rock close to the latter stream, about three miles distant from the ancient city of Angers, the former capital of Anjou—so celebrated in history, and formerly designated " La Ville Noire." Unlike the institution at Nantes, this asylum is appropriated exclusively to lunatics, and was originally an old chateau, first constructed in 1701 by a rich farmer-general of the public revenue. Many new buildings have, however, been recently added; and extensive alterations, with various improvements, are likewise now in progress; so that when all the contemplated structures shall be finished, it will exhibit quite a different aspect from the present, and better answer the purposes proposed. Until completed, strangers must therefore reserve their opinion; but judging from what I saw, and the plans of future operations obligingly exhibited for my inspection, the asylum of St. Gemmes-sur-Loire will doubtless become a first-rate institution.

The official machinery of this insane establishment is different from the institutions referred to previously, the resident physician being likewise superintending director, thus combining all the attributes of supreme medical and local administrative functions. There are no internes in the establishment, but the head of the asylum has a secretary and other subordinates to execute his orders and to manage details. Here, as in other asyla, there are also sisters of charity, who attend on the patients; but as all power centres in one individual, the discipline pursued was better than I have always noticed elsewhere.

During the year 1849, the movement amongst the insane patients was reported to be as follows:—

| | | | |
|---|---|---|---|
| Admitted ... ... ... ... | Males, 69 ... ... | Females, 57 ... ... | Total, 126. |
| Discharged, cured | Males, 32 ... ... | Females, 37 ... ... | Total, 71. |
| Died ... ... ... ... ... | Males, 39 ... ... | Females, 31 ... ... | Total, 70. |

Amongst the above seventy deaths, thirty-six males and twenty-six females, making a total of sixty-two individuals, were carried off by cholera; hence only eight patients of the entire number died from other causes. It is also worthy of notice, that nearly all the deaths by cholera occurred from the 28th of August to the 16th of last September, being the identical period when the recent epidemic prevailed most fatally in the British metropolis, the first seven days of the latter month having been designated by myself, in another publication, the " black week of 1849;" since 2298 human beings died in London from cholera alone during that short period of time, besides 885 by other causes.

On the day of my visit to St. Gemmes, the asylum contained 161 male patients and 179 females, making a total of 340 lunatics. Many of the inmates were incurable, their disease being of long continuance. Epileptics were numerous; general paralysis was common; a good many were idiots; several were cretins; and what is of rare occurrence in French asyla, two or three pellagreuse insane patients were pointed out for special observation. The women generally seemed more agitated than the male lunatics, the same as elsewhere observed, although not quite so noisy as I have sometimes witnessed. The men were more quiet, and certainly less talkative than the other sex, which peculiarity accords with general observation throughout France.

A very large proportion of the inmates were indigent persons, about thirty only, out of the total of 340 lunatics, being pensionary or paying patients, for whom the sums received varied from 500 to 1700 francs per annum. But from the information given me, the wish of the authorities, and especially of the medical director, seemed to be to render this institution a receptacle for the treatment of poor maniacs, rather than a place for receiving rich patients. The motive is highly creditable, and deserves recording, which I now do with much satisfaction.

No iron bars are seen in the dormitories, or any other apartment, excepting the cells for agitated patients, the windows throughout the establishment being protected by wire screens. The dormitories are well aired and spacious, the new portions being superior to the old; but as the latter will ultimately be replaced by improved structures, the defects now apparent will then disappear. The court-yards, although spacious, are defective of ornamental plots or flowers, as in most other asyla, and a *cabinet d'aisance* being placed in the centre of most, that object does not improve their appearance. Cleanliness and strict discipline pervade the whole establishment, and I could not avoid remarking the benefits accruing to the inmates by the orderly and systematic management pursued, which was creditable to the medical director, whose authority over every detail, whether lay or professional, was absolute; so different from the concurrent jurisdictions I have occasionally elsewhere observed.

During the peregrinations I made through the court-yards and dormitories, the number of patients then under restraint was as follows:—Male patients, twelve, all in camisoles, and two, if not three, also tied to their beds, having been very violent during the preceding night. The female patients in restraint were more numerous than the male lunatics, fifteen persons of the softer sex being also in strait-waistcoats, four of whom were likewise confined in bed, to keep them from injuring others or themselves. None, however, were shut up in any of the cells; and all the twenty-seven I saw under mechanical restraint were indigent patients.

Mental disease is rather more common in this district of France than in some neighbouring departments; intemperance and religion, but especially hereditary tendency, being a frequent cause of insanity amongst the native population. So great an influence, indeed, has hereditary tendency in the production of mental maladies, that in at least three-fifths of the cases met with, I was informed, such a predisposition may be traced. This makes a higher proportion than other physicians have usually reported; but it only renders the above fact more interesting. As a collateral illustration of the transmission to offspring of peculiar features in physical, as it is in mental organization, through many generations, I would mention the marked resemblance which several of the insane patients bore to the countenance and bodily frame of the ancient Romans; and one maniac in particular was pointed out to me, whose features, shape of head, and general contour of person, seemed exactly those of an old Roman. Indeed, if his bust had been taken in marble, it might almost pass for that of some antiquated senator or emperor. This will, however, appear less singular, when it is remembered, that the neighbouring district constituted formerly a very important Roman station, and was long occupied by that warlike people, who built a bridge over the Loire, near St. Gemmes, at a place still called "Pont de Cé," or the bridge of Cæsar. Besides which, the remains of an amphitheatre, and other interesting antiquities, are still visible in other localities near Angers.

Although the gardens of the asylum are frequently flooded in spring and autumn by inundations of the Loire, during summer, there is often a great want of water for necessary purposes. This deficiency of so essential an element, especially in an asylum for the insane, the director proposes to remedy by the erection of a steam engine, or a machine driven by horse power, to pump water from the adjacent river. This will prove a great adjunct to the establishment, and one which is very much required for the health and comfort of every resident.

Occupations of various kinds are carried on at the asylum by the inmates of both sexes, and much of the labour in the gardens is performed by patients. The alterations and improvements also in progress are advanced by their means; and in the adjoining farm —having upwards of forty acres, a similar system is pursued, the pigs, cows, and so forth, being often tended by insane patients, wherever practicable or judicious. A considerable part of the household work is also done by the female patients. Others are likewise occupied in knitting, sewing, mending, and making clothes; which employments are both profitable to the institution, as in part, likewise, to the individuals themselves. In fact, here, as in all French lunatic asyla, employing the inmates is considered essential, and a great adjunct in promoting recovery.

Having stated in a previous paragraph, that the offices of physician and director of the asylum are combined at St. Gemmes, before concluding this brief sketch of the above institution, it now only remains for me to say, Dr. Levincent fills these two important and responsible appointments, as he has, I believe, done for several years past, with much credit to himself and benefit to the institution. The task is, however, herculean, as he not only treats medically the various insane patients under his charge, registers their history and cases, but likewise superintends the feeding, clothing, and domestic arrangements of this extensive establishment, to say nothing of the financial and other departments under his immediate surveillance. Besides such laborious occupations, the numerous improvements and alterations now in progress likewise occupy much of his time and attention; but as Dr. Levincent is known to be a physician of energy and talent, he can, therefore, undertake official duties which to men otherwise constituted, would seem insuperable.

(*To be concluded in the January number.*)

---

